# Evaluation of the effectiveness of Aquatain, *Bacillus thuringiensis var. israelensis*, and Temephos on *Anopheles arabiensis* and *Anopheles stephensi* larvae in the laboratory and field settings

**DOI:** 10.1186/s13071-025-06765-4

**Published:** 2025-06-17

**Authors:** Sisay Dugassa, Tilahun Kebede, Bedri Abdulatif, Gudissa Assefa, Hiwot Solomon, Dejene Getachew, Kidane Lelisa, Araya Gebresilassie

**Affiliations:** 1 Aklilu Lemma Institute of Pathobiology, Addis Ababa University, Addis Ababa, Ethiopia; 2 Federal Ministry of Health, Malaria and Other Vector-Borne Diseases Prevention and Control Program, Addis Ababa, Ethiopia; 3 Department of Applied Biology, Adama Science and Technology University, Adama, Ethiopia; 4 Department of Biology, Dilla University, Dilla, Ethiopia; 5 Department of Zoological Sciences, Addis Ababa University, Addis Ababa, Ethiopia

**Keywords:** Larvicides, Temephos, Aquatain, *Bti*, *Anopheles stephensi*, *Anopheles arabiensis*

## Abstract

**Background:**

The main tools to control malaria vectors in sub-Saharan Africa are long-lasting insecticidal nets and indoor residual spraying. However, their sustainability is threatened by the emergence of insecticide resistances, behavioral avoidance and presence of outdoor biting mosquito populations. Thus, complementary interventions such as larval source management, which includes larviciding, are required to achieve better results in malaria vector control. This study aimed to evaluate the effectiveness of three larvicides (Aquatain AMF^®^, Temephos and *Bacillus thuringiensis var. israelensis*) against larvae of *Anopheles arabiensisi* and *Anopheles stephensi*.

**Methods:**

The tests on insectary colony and field-collected immature stages of the mosquitoes were conducted in the laboratory. For this, the third and fourth larval instars of *An. arabiensis* and *An. stephensi* were placed in trays measuring 50 cm × 40 cm, and larvicides were applied to the treatments while the control trays were left untreated. In addition, the larvicides were applied to selected natural habitats, and their effects on the reduction of the immature stages’ density were estimated.

**Results:**

In the laboratory, susceptible *An. arabiensis* showed mortality rates of 95% with Aquatain, 100% with *Bti* and 100% with Temephos, while *An. stephensi* showed 60% with Aquatain, 84% with *Bti* and 100% with Temephos. The percentage of larval mortalities among wild *An. arabiensis* collected from the field and exposed to Aquatain, *Bti* and Temephos were 97%, 100% and 100%, respectively, whereas those of *An. stephensi* were 74%, 99% and 100%, respectively. During the natural field study, the reductions in immature stages were as follows: 77%, 96% and 95% in Adama; 89%, 95% and 94% in Metahara; and 92%, 84% and 96% in Awash for Aquatain, *Bti* and Temephos, respectively.

**Conclusions:**

The three larvicides, Aquatain, Temephos and *Bti*, provided high levels of larviciding efficacies in both laboratory and field evaluations. Despite its effectiveness, Temephos caused the water to turn whitish and emitted a strong odor that made the community wary regarding the treated habitats. Therefore, we recommend using Aquatain in mosquito control programs as a complementary malaria vector control tool.

**Graphical Abstract:**

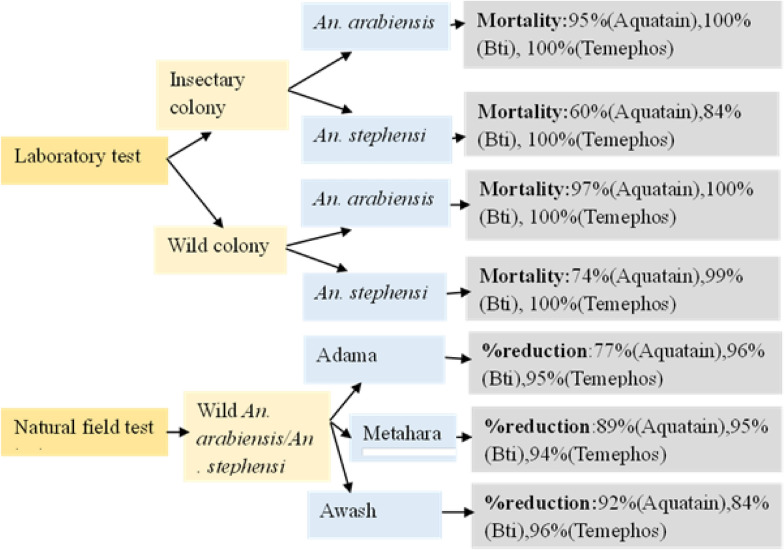

**Supplementary Information:**

The online version contains supplementary material available at 10.1186/s13071-025-06765-4.

## Background

Malaria remains a major public health problem in Ethiopia, with an estimated 5.1 million cases in 2022 [1]. It is also known to cause a significant financial burden on households. *Anopheles arabiensis*, a member of the *Anopheles gambiae* complex, is the main malaria vector in Ethiopia, while other malaria vectors such as *An. funestus*, *An. pharoensis* and *An. nili* are considered secondary malaria vectors [2]. In addition, *Anopheles stephensi*, a mosquito species previously associated with South Asia and the Persian Gulf, has recently been recorded in several localities in eastern, northeastern, southern and central Ethiopia [3–5]. Given that *An. stephensi* thrives in urban environments and is a highly competent vector for both *Plasmodium falciparum* and *P. vivax* [6], it thus constitutes a potential new threat to malaria control and hopes of elimination in the country.

Since 2005, malaria cases and deaths have been significantly reduced in Ethiopia following the scaling up of prompt diagnosis and treatment of cases with artemisinin-based combination therapy (ACT) and vector control tools, particularly long-lasting insecticidal nets and indoor residual spraying of insecticides [2]. These indoor interventions are one of the most effective methods of vector control in settings where mosquitoes are endophilic and endophagic. However, their marked contributions in the fight against malaria in several countries, including Ethiopia, have been undermined by multiple challenges, such as logistical difficulties [7], human behaviors that increase biting risk [8, 9] and insecticide resistance [10–12].

Some reports also showed that indoor vector control interventions such as insecticide-treated nets (ITNs) and indoor residual spraying (IRS) may cause changes in the biting and resting behaviors of mosquitoes, contributing to increased residual malaria transmission [13, 14]. Therefore, there is a pressing need to adopt complementary malaria vector control interventions to remove the threat to the sustainability of the currently adopted indoor vector control strategies such as ITNs and IRS. In this scenario, larval source management (LSM) can be an alternative supplement to effective indoor vector control tools.

Larval source management (LSM), which controls malaria vector populations by reducing the suitability of the mosquito larva habitat, is recognized as an effective additional tool for malaria control under certain conditions [15]. Larval source management has multiple benefits over the current indoor insecticide-based interventions, namely ITNs and IRS. Unlike household interventions, LSM has the potential to control vectors capable of evading indoor vector control interventions, including outdoor biting and outdoor resting, opportunistic biters, and early morning and early evening biting mosquitoes [16]. In addition, LSM may help to extend the useful life of insecticides against adult mosquitoes by reducing the size of the population being selected for resistance [17]. Several studies indicated that LSM has contributed to reductions in adult vector populations and malaria burden, especially where it has been integrated with other vector control tools [17–20]. Larviciding, through regular application of microbial or chemical insecticides to potential breeding habitats, is one of the common LSM methods used for targeting the larvae and pupae of mosquitoes [21]. Although larviciding with chemical agents was an important component of malaria vector control, only a few chemical larvicides are recommended for use in vector control, including insect growth regulators such as pyriproxyfen and organophosphates such as Temephos and Spinosad [22]. Biolarvicides from the microbial toxins *Bacillus thuringiensis var israeliensis* (*Bti*) and *Bacillus sphaericus* have also gained more ground as a tool for larviciding the breeding habitats of different mosquito species [23, 24]. Similarly, newer larvicide formulations, such as Aquatain, have been developed and tested in different ecological settings [25]. Aquatain is a new-generation product of monomolecular films and is a silicone-based larvicide. This larvicide was found to be effective against anopheline and culicine larvae under laboratory and field conditions [26–29].

Despite growing and renewed interest in LSM/larviciding in the control of malaria vectors in rural and urban settings in several countries in sub-Saharan Africa, larviciding has not been fully utilized in the fight against malaria vectors. This is mainly due to strong reliance on adulticides and less confidence in the efficacy of larvicides in particular and LSM in general. Furthermore, due to their significant adverse effects on other non-target species, chemical larvicides have received less attention. It is, therefore, necessary to generate local efficacy data on qualified and prequalified larvicide products to be incorporated to their fullest extent in the LSM program to supplement interventions targeting adult mosquitoes. This study was intended to supplement the available knowledge by comparatively testing the efficacy of different larvicides, Aquatain AMF® (https://aquatain.com/mosquito-control/aquatain-amf/), Temephos BASF Abates®500E (developed in Malasia in 2018 (https://www.mkhardware.com.my /pages_id/13613)) and *Bacillus thuringiensis var israeliensis (Bti)* (FourStar®Briquets of a solid form; produced by DBA FourStar Microbials LLC.1501 East Woodfed Road, #200W (https://www.centralmosquetocontrol.com/allproducts/fourstar/fourstar-briquet-180) in January 2019 and with expiry date of December 2023) to control *An. arabiensis* and *An. stephensi*. The findings of this study are crucial for guiding policymakers in including larviciding as a complementary strategy within vector control programs.

## Methods

### Test immature stages of mosquitoes

#### Susceptible immature stages for the in the laboratory condition

Third and fourth larval instars and pupae of insectary-reared *An. arabiensis* and *An. stephensi* from Aklilu Lemma Institute of Pathobiology (ALIPB) were used for the test under laboratory condition. During insectary rearing, mosquito eggs were housed in white enamel trays until larvae emerged. The larvae, at a density of 67 larvae/cm², were then transferred to plastic trays (measuring 20 cm × 15 cm × 5 cm) filled with de-chlorinated water (1.5 l). They were maintained under a 12:12 h light-dark photoperiod in accordance with established mosquito-rearing protocols [30]. These larvae were nourished with yeast, and their rearing medium was replaced every 3 days. Pupae were harvested using a glass beaker and placed in BugDorm cages measuring 30 cm on each side for adult emergence. Newly emerged adults were placed in mosquito cages at a temperature of 27 ± 3 °C and relative humidity of 70 ± 10%. They were provided with a sterile 10% sugar solution on cotton pads within petri dishes. The cotton was regularly moistened and changed daily. Female mosquitoes were also permitted to feed on a restrained rabbit three times weekly [approved by the Addis Ababa University Aklilu Lemma Institute of Pathobiology (AAU-ALIPB) Institutional Research Ethics Review Committee] to facilitate egg development and oviposition. Gravid female mosquitoes were encouraged to lay eggs on moist filter papers placed in rearing cages. These eggs were subsequently rinsed with deionized water onto larval trays to allow for hatching and the development of neonate larvae in the laboratory. Third- to fourth-instar larvae of the test mosquitoes were consistently utilized for larvicidal bioassays.

#### Wild immature stages for tests in the laboratory condition

For the tests on wild immature stages, feasible habitats with wild *Anopheles* larvae and pupae were selected from Awash, Metahara and Adama towns in eastern Ethiopia. The study sites were selected based on the information gathered from their respective health offices about malaria case occurrences, initial surveys or observations regarding the presence of suitable aquatic habitats for mosquito breeding, the abundance of malaria vector immature stages and accessibility for larvicide use, all aimed at ensuring effective intervention in malaria control. The larvae and pupae were collected from the same selected areas for the field testing. The larvae and pupae were collected using a standard dipper with World Health Organization (WHO) protocols and then carefully transferred into a 5-l plastic jar equipped with a handle and a ventilated cover for optimal air circulation. This specialized container was used to manage and transport the larvae and pupae. The collected larvae and pupae were sifted through dedicated clean cheesecloth and subsequently relocated to another plastic jar. Approximately 1 to 1.5 l water, supplemented with plant debris sourced from their natural breeding habitats, was introduced into the jar to sustain the larvae until their arrival at the insectary facility.

All larval instars and pupae collected from the field were transported to an enclosed insectary facility at the Aklilu Lemma Institute of Pathobiology (ALIPB) for further rearing into adults. This insectary is equipped with two secure doors—an outer double door at the entrance and individual doors with sealed glass windows for each unit within, ensuring containment and preventing mosquito escape. Upon arrival at the insectary, larvae were transferred into white enamel plastic trays. Using 1-ml micropipettes, larvae were carefully removed from their original water source in plastic containers and provided with a diet of baker's yeast within the larval tray. After a 5-min interval, the tray was gently spun to evenly distribute the yeast powder and prevent suffocation from any undiluted accumulation [31]. The larvae were reared to adults under the insectary condition described above in Sect. 2.1.1. The adults were also handled as described in Sect. 2.1.1 above [31]. Once it became gravid, each individual mosquito was placed in a 30 × 30 × 30-cm cage and was offered a water-filled petri dish or wet filter paper supported by cotton in a petri dish for egg-laying purposes.

Each mosquito was morphologically identified to species using a dissecting microscope. The eggs of mosquitoes of the same species, such as *An. arabiensis* and *An. stephensi*, were grouped together in labeled larval trays and housed in their designated rearing rooms. The experiments were carried out on the F_0_, F_1_ and F_2_ generations of the larvae and pupae.

#### Laboratory testing procedure

In the laboratory bioassay, the third and fourth larval instars of *An. arabiensis* and *An. stephensi* were thoroughly examined. To conduct the tests, three trays measuring 50 cm × 40 cm were specifically prepared for each larvicide under evaluation alongside an additional three trays designated for control purposes. The trays were carefully filled with distilled water up to their midpoints to ensure consistent test conditions across all experimental setups. Subsequently, 25 individual larvae were carefully transferred into each tray, ensuring an equal distribution of specimens for accurate observations. Aquatain, at a precise application rate of 1 ml per square meter, was then introduced into the test trays to assess its impact on larval mortality rates. Throughout the course of the experiment, mortality data were carefully recorded at 24-h intervals to track the larvicidal efficacy over time. Each bioassay was replicated for 3 days to account for potential variability and ensure robustness in the results obtained. This comprehensive investigative procedure was meticulously executed for both *An. arabiensis* and *An. stephensi* to ascertain the larvicidal effects of Aquatain under controlled laboratory conditions.

The same experimental procedures were applied to Temephos and *Bti* larvicides at specific concentrations: Temephos was administered at a rate of 0.25 ml per liter of water, while *Bti* was applied at a rate of 0.05 g per liter of water. These concentrations were selected based on their recommended dosages for effective larval control according to previous research and manufacturer guidelines. Each larvicide was thoroughly mixed with the water in the larval trays to ensure uniform distribution and consistent exposure levels for the mosquito larvae. The application rates were carefully calibrated to achieve optimal efficacy while minimizing potential environmental impacts. The three control trays were treated in a similar manner to the treatment trays, with the exception of not including the larvicides.

#### Field testing procedure

In the process of site selection, four ecologically comparable villages were identified based on shared characteristics such as topography, land cover and types of larval habitats across the study sites of Awash, Metahara and Adama. Of these villages, three were designated for the application of treatments, while one was kept as a control group for comparative analysis. The specific kebeles housing these villages were chosen through a meticulous selection process involving multiple criteria: The villages were selected based on information gathered from their respective health offices about malaria case occurrences, initial surveys or observations regarding the presence of suitable aquatic habitats for mosquito breeding, the abundance of malaria vector immature stages and accessibility for larvicide use, all aimed at ensuring effective intervention in malaria control.

Measuring mortality under field conditions poses a challenge due to the inability to quantify the total population of immature stages accurately. Therefore, in this study, estimated percent reductions of mosquitoes in different larval habitats were carefully examined to gauge the effectiveness of the interventions. The types of larval habitats identified and treated were largely alike, specifically cement tanks and cement water reservoirs (Additional file 1: Fig. S1). This approach allows for a comprehensive analysis of the impact on mosquito populations in their natural habitats, providing valuable insights into the efficacy of the treatments in real-world settings.

Before the larvicides were applied, the team conducted an estimation of larval densities per ten dips, serving as the baseline data for comparison. Following the application of the larvicides in the selected habitats, the densities of larvae were reassessed.

#### Data analyses

The mortality of the test sample was calculated by summing the number of dead larvae across all exposure replicates expressed as a percentage of the total number of exposed larvae. The mean percentage mortality of larvae was analyzed using SPSS version 25.0 software (SPSS Inc, Chicago, IL, USA). Data on percentage mortality of larvae were checked for normality by one-sample Kolmogorov-Smirnov Z test (K-S). As the data on percent mortalities did not conform to the normal distribution, a Kruskal-Wallis test was used to determine significant differences in the efficacies of different larvicides on mosquito larvae. Post hoc comparisons using Dunn’s method with a Bonferroni correction for multiple tests, adjusting the significance level per comparison to ensure that the familywise error rate was always below the level chosen (α = 5%), were done. In addition, one-way analysis of variance (ANOVA) was used to determine significant differences in the mean percent reductions of immatures (larvae and pupae) due to larvicides. Data were presented as mean ± SE, and *P* < 0.05 was considered to have statistical significance.

## Results

### Studies conducted in laboratory settings

An experiment conducted on susceptible *An. arabiensis* demonstrated notable differences in mortality rates among the exposed immature stages following exposure to Aquatain, *Bti* and Temephos. At 24 h post-exposure, the mortality rates were 95%, 100% and 100%, respectively (Table 1). Interestingly, Aquatain initially exhibited the lowest mortality rate at the 24 h only to effectively eliminate all larvae by the second day, showcasing its delayed but potent impact on the mosquito population. Similarly, the mortality outcomes for the exposed immature stages of susceptible *An. stephensi* were distinct. The mortality rates at 24 h post-exposure to Aquatain, *Bti* and Temephos were 60%, 84% and 100%, respectively (Table 1). These results underscore the varying effectiveness of the tested interventions against *An. stephensi*, with Temephos showing the highest mortality rate within the initial 24 h period.

**Table 1:** Efficacy of Aquatain, *Bti* and Temephos larvicides against insectary-reared and field-collected larvae of *Anopheles arabiensis*and *An. stephensi*

Treatments	Mean % mortality of *An. arabiensis* (insectary)	Mean % mortality of *An. stephensi* (insectary)	Mean % mortality of *An. arabiensis* (wild)	Mean % mortality of *An. stephensi* (wild)
Aquatain	95.11 ± 1.46^a^	60.44 ± 5.51^a^	97.33 ± 1.15^a^	73.77 ± 1.64^a^
*Bti*	100.00 ± 0.00^a^	83.56 ± 2.94^ab^	100.00 ± 0.00^a^	99.56 ± 0.44^b^
Temephos	100.00 ± 00^a^	100.00 ± 0.00^b^	100.00 ± 00^a^	100.00 ± 0.00^b^
Control	0.44 ± 0.44^b^	0.89 ± 0.59^c^	0.00 ± 0.00^b^	0.00 ± 0.00^c^

Mean values followed by the same letters across column are not significantly different (Dunn-Bonferroni post hoc test, *p* > 0.05)

Larvicidal efficacies of Aquatain, *Bti* and Temephos against field-collected wild *An. arabiensis* are presented in Table 1. Following a 24-h post-exposure period, the overall mortality rates were high at 97%, 100% and 100%, respectively (Table 2). Of note is the robust performance of all three treatments, showcasing their potent impact on controlling the mosquito population. Importantly, laboratory tests of Aquatain, *Bti* and Temephos against field-collected *An. stephensi* mosquito larvae revealed higher levels of larvicidal activity. At 24 h post-exposure, the overall mortalities were recorded at 74%, 99% and 100%, respectively (Table 1). Throughout the laboratory assessments, no or very few larval mortalities were recorded in the control trays (Table 1).

**Table 2:** Mean percentage reduction in effectiveness of three larvicides against mosquito larvae and pupae in the Adama site

Larvicides	Mean number of larvae and pupae/dip		% Reduction
	Pre-intervention	Post-intervention	
Aquatain	81.00 ± 11.37	18.67 ± 3.4	76.98 ± 1.02a
*Bti*	142 ± 24.98	6.00 ± 3.05	94.94 ± 2.81b
Temephos	151.67 ± 50.02	9.33 ± 4.98	93.94 ± 3.91b
Control	82.67 ± 14.40	82.00 ± 14.22	

Mean values followed by the same letters in columns are not significantly different (Tukey test, *p* > 0.05)

### Studies conducted in natural field settings

The results revealed substantial reductions in larval populations due to larvicidal effects of Aquatain, *Bti* and Temephos, resulting percent reductions of 77%, 96% and 95%, respectively, in the Adama site, as illustrated in Table 2. This careful pre- and post-treatment analysis provides a clear depiction of the efficacy of each larvicide in significantly reducing the mosquito larval populations within the specific study area.

In the specific locality of Metahara, the calculated estimated percentage reductions following the application of Aquatain, *Bti* and Temephos were found to be notably high, measuring at 89%, 95% and 94%, respectively. These results, detailed in Table 3, underscore the substantial efficacy of these larvicides in reducing mosquito populations in the Metahara site, providing valuable insights into their effectiveness in controlling larvae and pupae in this particular area.

**Table 3:** Mean percentage reduction in effectiveness of three larvicides against mosquito larvae and pupae in metahara town

Larvicides	Mean number of larvae and pupae/dip		% Reduction
	Pre-intervention	Post-intervention	
Aquatain	424.67 ± 76.46	44.67 ± 12.41	92.05 ± 1.19a
*Bti*	564.00 ± 240.67	26.0 ± 08.39	95.92 ± 0.52a
Temephos	315.00 ± 131.94	18.67 ± 13.24	96.36 ± 1.34a
Control	74.33 ± 24.88	89.00 ± 23.11	

Mean values followed by the same letters in columns are not significantly different (Tukey test, *p* > 0.05)

In Awash, significant reductions in mosquito populations were observed following the application of Aquatain, *Bti* and Temephos, with estimated percentage reductions of 92%, 84% and 96%, respectively. These findings, as presented in Table 4, highlight the considerable effectiveness of these larvicides in mitigating mosquito larvae and pupae in the Awash area. The data underscore the impactful role of these treatments in reducing the mosquito population, offering valuable insights into their efficacy in combating mosquito-borne diseases within this specific region.

**Table 4:** Mean percentage reduction in efficacy of three larvicides against mosquito larvae and Pupae in Awash town

Larvicides	Mean number of larvae and pupae/dip		% Reduction
	Pre-intervention	Post-intervention	
Aquatain	283.67 ± 82.95	22.00 ± 5.68	92.01 ± 0.98a
*Bti*	22.33 ± 9.52	3.67 ± 2.03	86.92 ± 6.71a
Temephos	222.33 ± 103.66	8.67 ± 4.37	96.58 ± 2.13a
Control	59.00 ± 28.43	60.00 ± 30.19	

Mean values followed by the same letters in columns are not significantly different (Tukey test, *p* > 0.05)

The aquatic habitats treated with Aquatain exhibited no noticeable alterations in color or odor. The application of the larvicide resulted in a uniform spread over the water surface, resembling a thin layer similar to a liquid. Visual documentation, depicted in Additional file 2: Fig. S2, showcases the condition after the larvicide application. While the photos may not distinctly reveal the changes, close physical examination revealed a substantial reduction in immature stages evidenced by the presence of deceased larvae floating on the water surface.

The aquatic habitats treated with *Bti* did not exhibit any noticeable changes in color or odor. Upon close inspection, the floating larvicide could be discerned, although not as prominently as with Aquatain. The post-larvicide application image is provided in Additional file 3: Fig. S3. Through physical observation, a significant reduction in immature stages was evident from the presence of deceased larvae floating in the water.

The aquatic habitats subjected to Temephos treatment exhibited notable alterations in both color and odor. Following the application of the larvicide, the water surfaces displayed a whitish hue accompanied by a distinct, pungent scent. Additional file 4: Fig. S4 provides visual documentation capturing the condition of the aquatic habitat after Temephos application. Unlike the scenarios involving Aquatain and *Bti*, physically identifying the deceased and floating larvae was difficult in the case of Temephos because of the nature of the treatment's impact on the water color.

## Discussion

The use of Temephos as a larvicide in Ethiopia has a longstanding history, with *Bti* emerging as a proposed alternative [32]. This study undertook a comprehensive comparative assessment of the relatively novel larvicide Aquatain alongside *Bti* and Temephos in both laboratory and field settings. Evaluations of these larvicides, conducted under laboratory conditions against both insectary-reared and field-collected *An. arabiensis* and *An. stephensi* populations, yielded consistent findings. Notably, the efficacy of Aquatain and *Bti* appeared to be somewhat diminished against *An. stephensi* compared to *An. arabiensis* in laboratory settings, irrespective of whether the mosquito strains were reared in controlled environments or collected from the wild.

In the field evaluation, various water storage containers such as barrels, geometric plastic reservoirs, overhead water containers and jerry cans in Awash Sebat Kilo and Metahara were devoid of *An. stephensi* larvae and pupae, contrasting with prior studies [11, 33, 34]. Conversely, cement-made water storage tanks and plastic drums proved to be conducive habitats for mosquito breeding. Interestingly, *Culex* and *Aedes* mosquito larvae were notably scarce in these environments, deviating from earlier reports [11, 33].

The absence of *An. stephensi* larvae and pupae in previously identified positive breeding sites could potentially be attributed to seasonal variations affecting mosquito productivity. Significantly, the field evaluation highlighted substantial reductions in immature stages across treated larval habitats, demonstrating consistent efficacy throughout various habitats and study areas.

The findings of this study underscore the varying degrees of effectiveness exhibited by the treatments against *An. stephensi*, with Temephos showcasing exceptional performance by achieving complete eradication of the exposed mosquitoes within the first 24 h. Aquatain exhibited varying rates of efficacy across different larval instars, demonstrating a swift impact on pupae and late fourth-instar larvae while displaying a slower effect on the first-, second- and early third-larval instars. Interestingly, this larvicide maintained the original color and odor of the water within the treated habitats. Mbare et al. [28] highlighted Aquatain's ability to sustain high levels of larval control for at least 6 weeks in open field systems. Their research further indicated that exposure to sub-lethal doses of Aquatain resulted in a reduction in the egg-laying capacity of female mosquitoes.

Moreover, standardized field experiments conducted by Mbare et al. [28] revealed that a single application of Aquatain Mosquito Formula (AMF) at a rate of 1 ml/m^2^ led to an 85% inhibition in adult mosquito emergence over a 6-week period (95% CI 82–88%). Their comprehensive evaluation concluded that the utilization of Aquatain presents a promising and innovative approach to larval control strategies targeting malaria vectors in Africa. Nonetheless, they emphasized the necessity of conducting field studies across diverse eco-epidemiological settings to ascertain the longevity of the Aquatain film for mosquito vector control and its potential integration into comprehensive integrated vector management programs.

The bacterial larvicide *Bti* demonstrated a rapid and significant impact on the immature stages of mosquitoes within a 24-h timeframe. Its efficacy appeared consistent across all developmental stages of the mosquitoes. Similar to Aquatain, *Bti* did not induce any changes in the color or odor of the water in the treated habitats, highlighting its non-intrusive nature. Notably, *Bti* exhibited a faster onset of action compared to Aquatain.

Studies focusing on the utilization of *Bti* for malaria vector control in sub-Saharan Africa, such as the research by Derua et al. [24], have underscored the effectiveness of these bacterial larvicide products in managing malaria vectors at low application rates. The outcomes of the present study align with research conducted in Iran, although employing varying concentration units of 512 and 4096 ppm for Bio-flash^®^ granules and powder formulations. In this context, *An. stephensi* larvae were found to be completely susceptible within 24 h post-treatment, as demonstrated by Gezelbash et al. [35].

Furthermore, the findings of this study corroborate laboratory test results involving different dosages of *Bti* VectoBac WDG against *An. stephensi* in Pakistan [36] and in India [37]. These studies highlighted the high susceptibility of *An. stephensi* larvae within 24 h post-treatment, providing additional support to the efficacy of *Bti* in swiftly controlling mosquito populations in diverse geographical regions. Temephos exhibited efficacy in both laboratory and field tests, demonstrating a swifter impact on early larval instars (first, second, third and early fourth larval stages) compared to pupae and late fourth-instar larvae. However, during field evaluations, communities expressed apprehension regarding the whitish color and potent odor of water treated with Temephos. The findings of this study align with research results from various countries [38–41].

Laboratory studies conducted in India and the southern regions of Iran indicated that larval bioassays with Temephos against *An. stephensi* larvae collected from the field showed susceptibility to the diagnostic dose of Temephos larvicide (0.25 mg/l) [38, 42]. Besides, a comprehensive review encompassing 56 pertinent studies on the effectiveness and operational feasibility of Temephos highlighted its effectiveness in controlling malaria vectors [43]. These collective findings underscore the consistent efficacy of Temephos across different regions and emphasize its role as a reliable tool in combating malaria vectors.

## Conclusions

The three larvicides, including Aquatain, Temephos and *Bti*, demonstrated high efficacy in combating mosquito larvae in both controlled laboratory settings and real-world field conditions. While Temephos exhibited notable effectiveness, its drawback lies in altering the water color to a whitish hue and emitting a strong odor, evoking concerns within the local communities residing near treated areas. This visual and olfactory impact underscores the importance of considering not only efficacy but also community acceptance in larvicide selection. In light of these findings, it is strongly recommended that Aquatain and/or *Bti* be prioritized in malaria control programs as complementary tools for managing malaria vectors. A sustained larvicidal activity of Aquatain over an extended period in field systems and *Bti*'s rapid onset of action at low application rates make them promising choices for integrated vector management strategies.

To ensure the continued success of larvicide-based interventions, it is imperative to emphasize the necessity of regular monitoring and evaluation. This ongoing assessment should focus on the efficacy, persistence, operational feasibility and community acceptance of these larvicides. By conducting thorough and consistent evaluations, stakeholders can optimize the use of these tools, tailor interventions to specific ecological contexts and enhance community engagement and support in malaria control efforts.

## Supplementary Information

Additional file 1: Figure S1. The two types of productive larval habitats selected for the field study: **A** Cement tankers, **B** cement water reservoirs.

Additional file 2: Figure S2. A picture of an aquatic habitat treated with Aquatain.

Additional file 3: Figure S3. A picture of an aquatic habitat treated with *Bti*.

Additional file 4: Figure S4. An illustration of an aquatic habitat treated with Temephos.

## Data Availability

No datasets were generated or analysed during the current study.
