# Supplementary figures and images for: Evaluation of the effectiveness of Aquatain, Bacillus thuringiensis var. israelensis, and Temephos on Anopheles arabiensis and Anopheles stephensi larvae in the laboratory and field settings
Source: Parasit Vectors. 2025 Jun 17;18:223. doi: 10.1186/s13071-025-06765-4 (PMC12172309; doi:10.1186/s13071-025-06765-4)

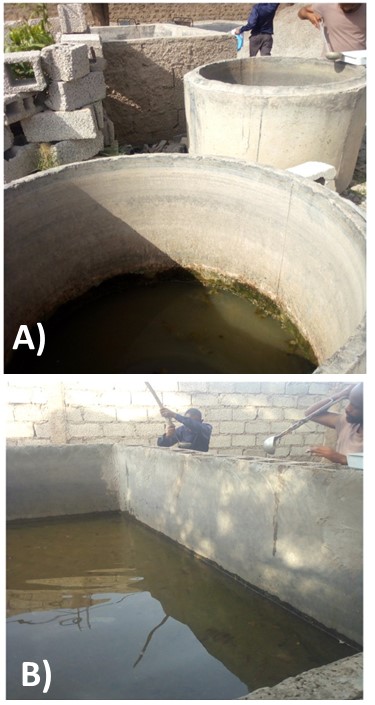

Supplement: Supplementary file 1 — Additional file 1: Figure S1. The two types of productive larval habitats selected for the field study: A Cement tankers, B cement water reservoirs. [file 13071_2025_6765_MOESM1_ESM.jpg]

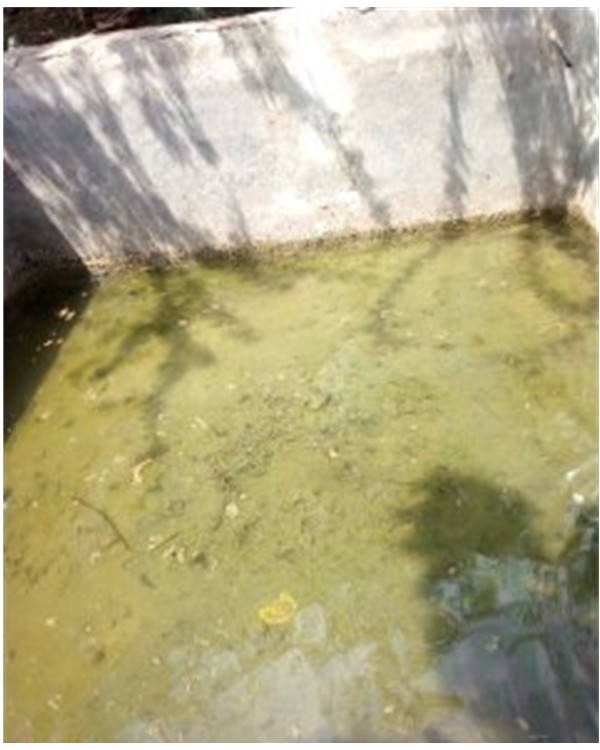

Supplement: Supplementary file 2 — Additional file 2: Figure S2. A picture of an aquatic habitat treated with Aquatain. [file 13071_2025_6765_MOESM2_ESM.jpg]

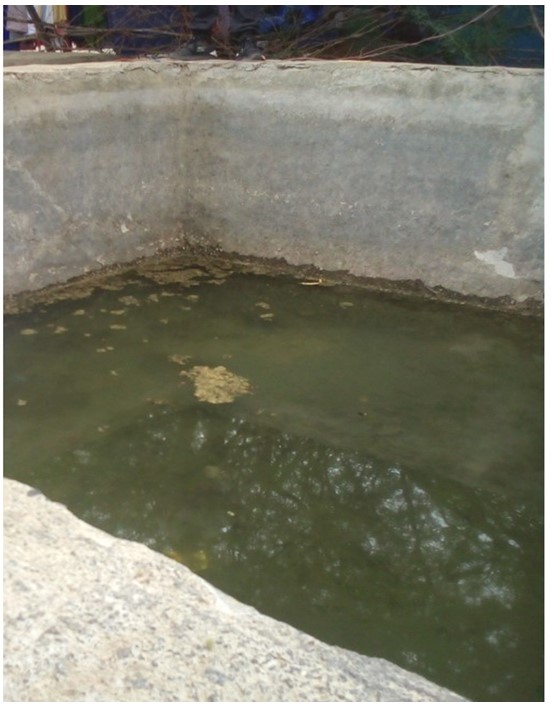

Supplement: Supplementary file 3 — Additional file 3: Figure S3. A picture of an aquatic habitat treated with Bti. [file 13071_2025_6765_MOESM3_ESM.jpg]

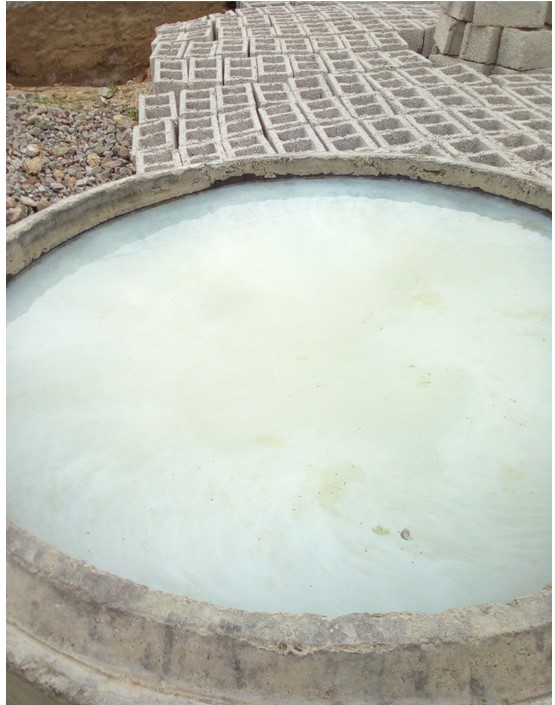

Supplement: Supplementary file 4 — Additional file 4: Figure S4. An illustration of an aquatic habitat treated with Temephos. [file 13071_2025_6765_MOESM4_ESM.jpg]
